# Mandibular incisors angulation and position among various vertical facial patterns: An artificial intelligence-based cephalometric study

**DOI:** 10.6026/973206300210113

**Published:** 2025-02-28

**Authors:** Nada Mohammed Aloufi, Faris Saleh Al Qazlan, Ahmed Ali Alfawzan

**Affiliations:** 1 Department of Orthodontic and Pediatric Dentistry, College of Dentistry, Qassim University, Kingdom of Saudi Arabia

**Keywords:** Artificial intelligence, mandibular incisors, facial patterns

## Abstract

The position and inclination of the mandibular incisors are critical factors in orthodontic diagnosis, treatment and retention. Therefore, it is of interest to evaluate the position and angulation of mandibular incisors across various vertical growth patterns. One hundred and three cephalograms from untreated patients were analyzed using the artificial intelligence-based Webceph software. The mandibular incisor position and angulation of the vertical group exhibited the highest values, while the horizontal group demonstrated the lowest values. Thus, mandibular incisors exhibit greater proclination and protrusion in the vertical pattern group, while displaying increased retroclination and retrusion in the horizontal pattern group.

## Background:

The mandibular incisors' position and inclination are essential diagnostic indicators in orthodontics and are critical factors in orthodontic treatment planning, stability and retention [1]. Relation between function and shape, can be applied to orthodontic patients through skeletal compensations and dentoalveolar compensations [2]. In addition to other factors, incisors inclination and position have a significant effect on incisors alignment. Mandibular incisors crowding increased in subject with retroclined lower incisors [3]. Many researches discussed the compensation of dentation to different stage craniofacial growth and development [2, 4]. The dental compensation in both vertical and sagittal is a part of achieving normal occlusion process. Researches revealed a difference in mandibular incisors angulation among various sagittal and vertical growth pattern [5, 6]. Lips prominence is affected by maxillary and mandibular incisors inclination and position [7]. Vertical growth pattern has significant relation with mandibular incisor angulation [5, 8]. Mandibular incisors are more poclined and extruded in subject with vertical growth pattern than subjects with horizontal growth pattern [8]. Therefore, it is of interest to investigate the correlation of mandibular incisors position and angulation with different vertical growth patterns.

## Materials and Methods:

A total of one hundred and three cephalograms of untreated patients (45 females and 58 males; mean age 20 ± 2.3 years) were collected from the orthodontic clinic, Riyadh Specialist Dental Center, KSA. The Regional Research Ethics Committee, Qassim Province, approved this study (Code#607/46/4843).A sample of 97 cephalograms was calculated using a 95% confidence level and a 5% margin of error, a total of 103 cephalograms included in this study. The inclusion criteria included: clear lateral cephalometric radiographs, mature patients, skeletal Class I relationship as determined by the ANB value (2° ± 2°) and no history of orthodontic treatment. To assess errors of measurement, twenty radiographs were chosen at random and analyzed for this study variable. The concordance correlation coefficient test did not reveal any significant differences when the same observer repeated the tracing after 2 weeks. One examiner used automatic A.I.-driven Webceph software (South Korea) to measure all of the cephalometric parameters. Table 1 shows the variables of this study. The sample was categorized into horizontal, balanced and vertical growth patterns based on the Frankfort plane and the mandibular plane angle (FMA) mean value, as suggested by Tweed [9]. An FMA greater than 30° was interpreted as a vertical growth pattern, whereas an FMA lower than the typical range (22° to 28°) was interpreted as a horizontal growth pattern. Consistency with the assumption of homogeneity of variances using Shapiro-Wilk and Levene's test was evaluated before any group comparisons. An ANOVA test was conducted to evaluate the differences in means across the groups and Tukey's post hoc test was utilized to assess the significance of these mean differences. SPSS version 22.0 was used for the statistical analysis. For P <.05, the variances were regarded as statistically significant. The differences were considered statistically significant for P <.05.

## Results:

The sample for the study included 103 skeletal Class I patients (ANB: 2° ± 2°), categorized based on their vertical growth patterns: 37 were classified as balanced (ages 18 to 27 years, mean age 18.7 ± 2.1), 30 as horizontal (ages 19 to 25 years, mean age 18.9 ± 3.4) and 36 as vertical growth pattern (ages 18 to 28 years, mean age 19.9 ± 1.8).The mean values of the lower incisors' position and angulation for different vertical growth pattern groups are shown in Table 2. The means of mandibular incisor position and angulation of the vertical group was the highest, whereas it was the least for the horizontal group. ANOVA result showed significant differences in mandibular incisor angulation between the three groups (P <.05). When comparing the mean of mandibular incisors position (Linc-NB distance) of vertical group to balanced and horizontal growth pattern, the Tukey test indicated that the mean mandibular incisors position of the vertical group did not significantly differ from balanced and horizontal groups (P > .05). The mean mandibular incisors position (Linc-NB distance) did not vary considerably between balanced and horizontal group (P > .05) Table 3. The Tukey test indicated that the mean angulation of mandibular incisors in the vertical group was significantly greater than that in the balanced and horizontal groups (PP<.05) for both IMPA and Linc-NB angles. The mean angulation of the mandibular incisors did not exhibit a significant difference between the balanced and horizontal groups (P > .05), as seen in Table 4.

## Discussion:

This study involved cephalometric analysis and tracing utilizing digital artificial intelligence. Digital tracing enhances the detection of landmarks, leading to improved precision of cephalometric analysis and superimposition [10]. The inclination and position of the mandibular incisors have a significant role in orthodontic diagnosis, treatment planning, aesthetics and stability. Excessive proclination of mandibular incisors negatively impacts the supporting periodontal tissues and aesthetics [3, 11]. Our study revealed no statistically significant correlation between the position of the mandibular incisors and the vertical pattern. These findings coincide with those of Amin Erum *et al.* [12] and Jabbar *et al.* [13], who similarly did not identify any significant correlations between these variables. Similar to our study, Mouakeh [14] observed that in Class III individuals with reduced vertical facial height, the mandibular incisors only displayed a little amount of retrusion. A direct relation between the mandible and the incisors can be achieved through the utilization of mandibular incisors to mandibular plan angle. This method eliminates the need for cranial references [15]. Germec-Cakan *et al.* [16] pointed out that to prevent relapse, it is essential to avoid resolving of crowding by labial dental segment expansion, particularly in individuals with dolichofacial facial pattern. Moreover, Hurtado *et al.* [17] found that subjects with dolichofacial biotypes had an increased dental inclination compared to other facial biotypes. This study found a significant correlation between incisors proclination and vertical pattern. Similar to our findings, Hernandez *et al.* [11] Found that subjects with upward rotated mandibular plane have more retro lined mandibular incisors. As opposed to average and horizontal patients, the movement of mandibular incisors should be restricted in vertical pattern patients [18]. Excessive expansion of mandibular labial segment may cause periodontal complications, bone fenestration and other iatrogenic disorders [19].

## Conclusion:

Mandibular incisors are more protruded and proclined in subjects with vertical growth pattern. Hence, care should be taken during orthodontic treatment to avoid over labial expansion of mandibular incisors. Further, excessive orthodontic proclination of mandibular incisors has iatrogenic (illness caused by medical examination) periodontal complication.

## Figures and Tables

**Table 1:** Measurements used in this study

**Variable**	**Measurement**	**Norms**	**Description**
Vertical Growth Pattern	FMA	22°±6°	The angle formed by the Frankfort plane (FH) and themandibular plane (MP)
Incisors Position	Linc-NB distance	4mm	The linear distance measured from the most prominent the mandibular incisor edge perpendicular to NB line
Incisors Angulation	IMPA	90°±5°	The angle formed by the long axis of the mandibular incisor to Go-Me
	Linc-NB angle	25°	The angle formed by the long axis of the mandibular incisor and NB line

**Table 2:** Means of mandibular incisors position and angulation of the three Groups

**Variable**	**Balanced Group**	**Horizontal Group**	**Vertical Group**	**P**
Linc-NB distance	3.6 mm	3.4mm	4.2 mm	0.25
IMPA	91°	87°	98°	0.004
Linc-NB angle	26°	23°	28°	0.002

**Table 3:** Comparison of mandibular incisors position (Linc-NB distance) among the groups

**Growth pattern**	**P**
Vertical vs Horizontal	0.063
Vertical vs Balanced	0.124
Balanced vs Horizontal	0.232

**Table 4:** Comparison of mandibular incisors angulation among the groups

**Growth pattern**	**IMPA**	**Linc-NB**
	**P**	**P**
Vertical vs Horizontal	0.017	0.043
Vertical vs Balanced	0.002	0.036
Balanced vs Horizontal	0.421	0.352
